# The histidine-rich glycoprotein A1042G polymorphism and recurrent miscarriage: a pilot study

**DOI:** 10.1186/1477-7827-12-70

**Published:** 2014-07-27

**Authors:** Evangelia Elenis, Karin E Lindgren, Helena Karypidis, Alkistis Skalkidou, Frida Hosseini, Katarina Bremme, Britt-Marie Landgren, Lottie Skjöldebrand-Sparre, Anneli Stavreus-Evers, Inger Sundström-Poromaa, Helena Åkerud

**Affiliations:** 1 Department of Women’s and Children’s Health, Uppsala University, Uppsala, Sweden; 2 Centre for Clinical Research, Värmland County Council, Karlstad, Sweden; 3 Department of Clinical Sciences, Division of Obstetrics and Gynaecology, Karolinska Institutet, Danderyd Hospital, Stockholm, Sweden; 4 Department of Women’s and Children’s Health, Karolinska Institutet, Stockholm, Sweden; 5 Department of Clinical Sciences, Intervention and Technology, Karolinska Institutet, Karolinska University Hospital Huddinge, Stockholm, Sweden

**Keywords:** Histidine-rich glycoprotein, Recurrent miscarriage, Single nucleotide polymorphism

## Abstract

**Background:**

Histidine-rich Glycoprotein (HRG) has previously been shown to have an impact on implantation and fertility. The aim of this study was to investigate if there is an association between the HRG A1042G single nucleotide polymorphism (SNP) and recurrent miscarriage.

**Methods:**

The study was designed as a case-control study and the women were included at University Hospitals in Sweden. 186 cases with recurrent miscarriage were compared with 380 pregnant controls with no history of miscarriage. Each woman was genotyped for the HRG A1042G SNP.

**Results:**

The results indicated that the frequency of heterozygous HRG A1042G carriers was higher among controls compared to cases (34.7% *vs* 26.3%; p < 0.05). In a bivariate regression analysis, a negative association was found between recurrent miscarriage and heterozygous A/G carriers both in the entire study population (OR 0.67, 95% CI 0.45 - 0.99; p < 0.05) as well as in a subgroup of women with primary recurrent miscarriage (OR 0.37, 95% CI 0.16 - 0.84; p < 0.05). These results remained even after adjustment for known confounders such as age, BMI and thyroid disease (OR 0.36, 95% CI 0.15 - 0.84; p < 0.05).

**Conclusions:**

Women who are heterozygous carriers of the HRG A1042G SNP suffer from recurrent miscarriage more seldom than homozygous carriers. Thus, analysis of the HRG A1042G SNP might be of importance for individual counseling regarding miscarriage.

## Background

Recurrent miscarriage was defined as three or more consecutive pregnancy losses before 20 weeks of gestation [1]. Since then several guidelines have been launched but their definitions vary regarding the number as well as the sequence of preceding miscarriages; recurrent miscarriage is defined as two or more versus three or more consecutive pregnancy losses according to ACOG [2] and ESHRE [3] as well as RCOG [4] respectively. However the definitions from ASRM [5] and NVOG [6] (two or more miscarriages) do not contain the word consecutive.

It affects approximately 1-5% of fertile couples [1,7] and the risk of recurrence increases with maternal age and number of consecutive pregnancy losses [8]. The reason for recurrent miscarriage on an individual basis is often unclear, but parental and fetal chromosomal aberrations, uterine abnormalities and immunological, endocrinological and thrombophilic disorders play important role [3]. Although the condition is common, underlying causes can only be determined in about 50% of the cases [9]. Consequently, remaining cases are classified as idiopathic with a presumably heterogeneous and multifactorial etiology [1]. Current research in this patient group has focused on defects related to implantation and/or placentation as well as embryogenesis.

Angiogenesis, which refers to the formation of new blood vessels from preexisting ones, is required during embryonic development as well as in implantation and placentation. Based on this hypothesis several studies have been performed examining the potential contribution of genetic polymorphisms encoding angiogenic mediators (such as VEGF, eNOS, p53 etc) [10,11]. One of the endogenous regulators of angiogenesis circulating in high levels in plasma is Histidine-rich Glycoprotein (HRG). The protein is synthesized in the liver and it is either transported as a free protein or stored in α-granules of platelets and released after thrombin stimulation [12]. The human HRG gene is localized at chromosome 3 in position 3q28-29 and it encodes a 507 amino acid long multi-domain protein [13]. Structurally, HRG consists of three distinct domains: a NH2-terminal part with two cystatin-like domains, a central histidine/proline-rich domain and a COOH-terminal domain [13]. It interacts with a number of different biological pathways such as regulation of chemotaxis and focal adhesion of endothelial cells, cytoskeletal organization during vessel formation and immune complex formation. Its action is mediated by a variety of ligands including heparin/heparan sulfate, thrombospondin, plasminogen and divalent metal ions [13].

HRG might be involved in several of the processes of importance for a pregnancy to occur but the exact role of HRG in fertility is not yet fully described. We have previously shown that HRG exists in the reproductive system as well as in the embryo [14] and it is involved in the hypercoagulability and the angiogenic imbalance seen in early onset preeclampsia [15]. HRG has been reported to exist as at least ten naturally occurring single nucleotide polymorphisms (SNPs) [16,17]. One of the SNPs, the HRG C633T SNP, has been postulated to be of relevance for implantation and a successful pregnancy [18] and there is an increased occurrence of the homozygous T/T genotype in patients with primary recurrent miscarriage [19].

In another SNP of the gene, named HRG A1042G, an adenine (A) nucleotide is replaced by a guanine (G), which results in a change from histidine to arginine at amino acid position 340 in the protein. HRG A1042G is localized in the histidine/proline (His/Pro) rich region, which is known to mainly regulate the anti-angiogenic capacity of HRG [20]. No study has, however, been performed yet examining its importance in pregnancy. Since adequate regulation of angiogenesis is well known to be of relevance for implantation and placentation, as well as for embryogenesis, the aim of this study was to investigate if there is an association between the HRG A1042G SNP and recurrent miscarriage.

## Methods

### Study population

The study was designed as a case-control study. Cases (n = 186) were recruited from the Department of Obstetrics and Gynaecology at Uppsala University Hospital, Karolinska University Hospital, Huddinge University Hospital and Danderyd University Hospital, Sweden. Eligible cases with a diagnosis of recurrent miscarriage, defined as three or more verified consecutive miscarriages in the first or second trimester of pregnancy (5-21 completed weeks of gestation), were identified in the out-patient registers of the participating clinics and invited to participate in the study. The women were included between April 29, 2009 and June 30, 2010 sometimes years after the initial incidence. Women with known risk factors for recurrent miscarriage, such as systemic lupus erythematosus, diabetes mellitus type 1, severe thrombophilia and major chromosomal aberrations were not included in the study.

The control subjects (n = 380) were randomly chosen from the Uppsala University Hospital biobank of pregnant women. Since May 31, 2007, all women aged 18 and older attending the second trimester (16-19 weeks of gestation) routine ultrasound scan at Uppsala University Hospital have been approached for inclusion in this biobank, and inclusion to our study was ongoing until June 30, 2010. All control subjects were followed until delivery and only term pregnancies fulfilled the inclusion criteria. In the control group, no woman had a history of miscarriage or was treated with anti-thrombophilic medication (such as Low Molecular Weight Heparin or Aspirin) and 75% had at least two spontaneous pregnancies, including the ongoing pregnancy, resulting in a term (≥37 weeks) birth of a live infant. Thirty-two percent of our cases had already a child before recruitment. Cases and controls differed only in their obstetric record but had otherwise similar inclusion and exclusion criteria.

Both cases and controls attended a brief health examination including measurements of weight and height and answered standardized questions on reproductive history. Furthermore, we reviewed the medical records to obtain relevant information on pregnancy outcomes, health problems and medication. According to routine clinical procedures, TSH-levels were analysed in all women when diagnosed with recurrent miscarriage. Among cases hypothyroidism was defined as TSH above the current defined upper limit of the reference range at the different hospitals. Pregnant controls at high risk for thyroid disease were subjected to selected TSH screening according to local guidelines based on international recommendations. This case-finding procedure was applied in first or early second trimester of pregnancy, before routine ultrasound scan and inclusion in the study. Since ethnicity affects genetic variation, the population distribution was then investigated on our study group. It was found that 94.6% (176/186) of cases and 95.5% (363/380) of controls were of Caucasian origin and the frequency did not differ statistically between the two groups (p = 0.89). All the analyses that follow are performed in an ethnically mixed group.

A post hoc power calculation, where a Chi-square test with an alpha-value of 0.05 was used, gave a post hoc power of 0.97.

The study was approved by the Regional Ethical Review Boards in Uppsala and Stockholm, Sweden. Informed consent was obtained from all women included in the study. No reimbursement was given to participating patients.

### Blood sample collection

Blood samples were collected in EDTA-containing tubes and centrifuged at 1500 *g* for 10 min. Plasma and buffy coat were separated, transferred to new tubes and stored at -20°C.

### SNP analysis

Genomic DNA was extracted from buffy coat using QIamp DNA Blood Maxi kits (Qiagen, venlo, the Netherland). The samples were genotyped for the HRG A1042G (rs2228243), using the TaqMan SNP Genotypning Assay (Applied Biosystems, Foster city, CA, USA). Briefly, polymerase chain reactions were performed in a 96-well plate in total volume of 25 μl for each well. Each reaction consisted of 1xTaqMan Universal PCR Master Mix (PCR buffer, ROX passive reference dye, dNTPs and AmpliTaq Gold polymerase), 1× SNP Genotyping Assay (sequence-specific forward and reverse primers to amplify the polymorphic sequence of interest, ie HRG exon 5, Taq man MGB probes labelled with VIC dye to detect allele 1 sequence and with FAM to detect allele 2 sequence) and 10 ng of genomic DNA. Cycling conditions were initiated for 10 min at 95°C followed by 40 cycles of 15 s at 92°C and 1 min at 60°C. Real-time fluorescence detection was performed. Sequence Detection system Software (Applied Biosystems) was used to plot fluorescence (Rn) values based on the signals from each well. The plotted fluorescence signals indicated which alleles were present in each sample.

### Statistical analysis

Demographic and clinical characteristics were compared between cases and controls or genotype groups using Student’s t-test, Mann-Whitney U test, Chi square test and Kruskal-Wallis test. At first, a logistic regression analysis was performed examining the association between HRG A1042G SNPs and recurrent miscarriage. Secondly, in a subgroup analysis where only women with primary recurrent miscarriage were included (defined as recurrent miscarriage and no known children either before or after diagnosis), a comparison with controls was performed using Student’s t-test and Chi-square test. A logistic regression model was composed. A number of possible confounders were considered for inclusion in the regression model; age, pre-pregnancy smoking, BMI, thyroid disease and genotype. Only variables with a possible association with exposure and outcome (p-value < 0.25) were entered into the final model. Adjusted odds ratios (AOR) for recurrent miscarriage were calculated in logistic regression analyses including the following variables: maternal age as completed years at the first pregnancy or first miscarriage (two categories, ≤ 35 years or > 36 years), body mass index (BMI, kg/m^2^) defined as BMI recorded at inclusion (cases) or BMI at first antenatal visit (controls) (two categories, ≤ 30 kg/m^2^ or > 31 kg/m^2^), smoking during pregnancy (yes/no) defined as smoker during ≥ 1 pregnancy ending with miscarriage (cases) or smoker at first visit to the prenatal center in gestational week 10 (controls), and genotype (two categories, heterozygous compared to homozygous HRG A1042 SNP and HRG 1042G SNP). A p-value < 0.05 was considered as significant. All statistical analyses were performed using the Statistical Package for the Social Sciences (SPSS) 20.0 for Windows software pack (SPSS, Chicago, IL).

## Results

### Background characteristics and HRG genotype

Demographic data and clinical characteristics concerning cases (n = 186) and controls (n = 380) are shown in Table 1. There was no difference in age between cases and controls. BMI was higher among cases (24.7 *vs* 23.8; p < 0.05), and as expected, hypothyroidism was more common in women with recurrent miscarriage (8.6% *vs* 3.2%; p < 0.01). All women were genotyped according to the HRG A1042G SNP and the frequency of heterozygous (A/G) carriers was significantly higher among controls compared to cases (34.7% *vs* 26.3%; p < 0.05, Table 1).

**Table 1:** Background characteristics of the study population

	**Cases (n = 186)**	**Controls (n = 380)**
*Age, years*	30.1 ± 5.7	30.1 ± 5.7
BMI, kg/m2*	24.7 ± 4.8	23.8 ± 4.0
Smokers, n (%)*	34 (18.3%)	41 (10.8%)
Hypothyroidism, n (%)**	16 (8.6%)	12 (3.2%)
***HRG A1042 genotype***		
*A/A*	128 (68.8%)	231 (60.8%)
A/G*	49 (26.3%)	132 (34.7%)
*G/G*	9 (4.8%)	17 (4.5%)

Values are mean ± standard deviation; n, number of women; BMI, body mass index (missing data in 1 subject) at the recruiting time for cases or at the first antenatal visit for the controls; Pre-pregnancy smokers, smoker during ≥ 1 pregnancy ending with miscarriage for the cases or at first visit to the prenatal center in gestational week 10 for the controls.

*p < 0.05, **p < 0.01.

Among all the 566 women genotyped for HRG A1042G SNP, 359 (63.4%) of them were homozygous for adenine (A/A), 181 (32%) were heterozygous (A/G) and 26 (4.6%) were homozygous for guanine (G/G) (Table 2). The number of homozygous and heterozygous carriers of the SNP was in accord with the Hardy-Weinberg equilibrium. BMI, age and smoking did not differ in relation to genotype. Hypothyroidism was more prevalent among the homozygous G/G carriers (Table 2).

**Table 2:** Demographic data and clinical characteristics of the study population according to the HRG A1042G genotype

	**A/A (n = 359)**	**A/G (n = 181)**	**G/G (n = 26)**
*Age, years*	30.0 ± 5.8	30.2 ± 5.8	30.7 ± 5.0
*BMI at first antenatal visit, kg/m*^*2*^	24.4 ± 4.4	23.6 ± 4.0	23.9 ± 4.8
*Pre-pregnancy smokers, n (%)*	45 (12.5%)	27 (14.9%)	3 (11.5%)
Hypothyroidism, n (%)*	16 (4.5%)	8 (4.4%)	4 (15.4%)
Recurrent miscarriage*	128 (35.7%)	49 (27.1%)	9 (34.6%)
Recurrent miscarriage without child*	32 (12.2%)	7 (5%)	4 (19%)

Values are mean ± standard deviation; n, number of women; BMI, body mass index; Pre-pregnancy smokers for cases or smoker at first visit to the prenatal center in gestational week 10 for the controls.

*p < 0.05.

### Genotype and risk for recurrent miscarriage

At first, a logistic regression analysis in the entire study population was performed where an association between genotype and the condition of recurrent miscarriage was identified. The heterozygous A/G carriers presented the lowest risk compared to A/A or G/G carriers (OR 0.67, 95% CI 0.46 - 0.99; p < 0.05).

In order to study recurrent miscarriage as a reason for sub-fertility, a statistical subgroup was constructed. This group was defined as ‘diagnosed with primary recurrent miscarriage’ (no known children either before or after diagnosis) [19,21], and consisted of 43 women who were compared to controls. Age, BMI and smoking did not differ between the groups, but hypothyroidism was more prevalent (p < 0.001) among cases. The frequency of heterozygous A/G carriers was significantly lower among controls than cases (34.7% *vs* 16.3%, p < 0.05). To further investigate the impact of the HRG A1042G SNP on recurrent miscarriage a logistic regression analysis was performed on this subgroup of women. A significant association between women carrying the heterozygous HRG A1042G SNP and recurrent miscarriage was identified, with a decreased risk of recurrent miscarriage among these women (OR 0.37, CI 95% 0.16-0.84; p < 0.05). As expected, hypothyroidism was associated positively with recurrent miscarriage (OR 4.97 CI 95% 1.76-14.02; p < 0.01). Other known confounders such as age, smoking and BMI were not associated with recurrent miscarriage (Table 3). The women with the heterozygous HRG A1042G SNP were furthermore shown, after performing a logistic regression analysis adjusted for the above mentioned known confounders, to be protected from recurrent miscarriage (OR 0.36, 95% CI 0.15-0.84; p < 0.05).

**Table 3:** Factors associated with recurrent miscarriage in association with HRG A1042G genotype in a subgroup analysis (cases include only women with primary recurrent miscarriage)

	**Odds ratio**	
	**Unadjusted**	**Adjusted**
***Age***		
*≤35 years*	1	1
*≥36 years*	1.31 (0.63-2.71)	1.40 (0.65-2.96)
***BMI, kg/m***^***2***^		
*≤30*	1	1
*≥31*	1.86 (0.77-4.48)	1.99 (0.80-4.95)
***Smoking***		
*No*	1	1
*Yes*	2.19 (0.98-4.89)	2.37 (1.04–5.44)^*^
***Hypothyroidism***		
*No*	1	1
*Yes*	4.97 (1.76-14.02)^**^	4.63 (1.61–13.36)^**^
***HRG A1042G genotype***		
*A/A or G/G*	1	1
*A/G*	0.37 (0.16-0.84)^*^	0.36 (0.15-0.84)^*^

BMI, body mass index; Pre-pregnancy smokers for cases, or smoker at first visit to the prenatal center in gestational week 10 for controls; HRG A1042G genotype refers to either heterozygous carriers (A/G) or homozygous carriers (A/A) or (G/G).

Adjusted for age, BMI, smoking and thyroid disease.

^*^p < 0.05, ^**^p < 0.01.

## Discussion

In the present study, a possible association between the HRG A1042G SNP and recurrent miscarriage has been investigated. We found that among women with recurrent miscarriage there was a decreased occurrence of heterozygous HRG A1042G SNP carriers and the association was even stronger in the group with primary recurrent miscarriage. Previous computational studies have shown that the amino acid shift in position 340 related to that specific SNP is predicted to be benign or tolerated according to Polyphen-2 and SIFT respectively (position-specific independent count score 0.227 and tolerance index 0.34) [22]. The latter is partly in agreement with our finding that heterozygous A/G carriers have a lower occurrence of recurrent miscarriage compared to homozygous A/A. Further in vitro studies are required to investigate the underlying mechanisms and the mediators involved in the increased risk of recurrent miscarriage in homozygous G/G compared to heterozygous A/G carriers. It should nevertheless be reminded that recurrent miscarriage is thought to be a syndrome of multifactorial origin and not a Mendelian disease. Therefore it might be related to various genetic polymorphisms and their combinations and not solely on a single one. Our results are in agreement with the theory of heterozygote advantage according to which heterozygous carriers present a selective advantage in viability and reproductive fitness over homozygous in natural populations [23]. Although the hypothesis of “heterozygosity-fitness” correlation has mainly been associated with disease resistance in evolutionary biology, some recent reports describe possible associations between specific polymorphisms and fertility [24,25]. A similar reproductive advantage among HRG heterozygous carriers cannot be ruled out.

The establishment of a pregnancy requires adequate regulation of angiogenesis, coagulation, as well as regulation of immunological pathways [3]. Development of a normal well functioning vasculature in the uterus and placenta requires cooperation between different cell types and various growth factors in the processes of implantation, embryo development and placentation. The exact coordination of these processes is complex, which is further indicated by the fact that a defect in one single factor that may interact with others might induce pregnancy failure [26]. In microarray analyses on endometrial biopsies [27] as well as decidual samples [28] from women with recurrent miscarriage, it has been shown that genes from different functional areas have been inadequately regulated, with the most important genes being related to control of cell adhesion, cell migration and angiogenesis. In addition, studies evaluating the role of gene polymorphisms coding for the most known mediators of angiogenesis such as VEGF, eNOS and p53 tend to find positive associations to the condition [10,11].

We have previously shown that HRG might be of importance in early pregnancy and this is supported by our prior results indicating an association between the presence of HRG in placenta and risk of preeclampsia during pregnancy [15], as well as between the HRG C633T SNP and pregnancy outcome among patients undergoing *in vitro* fertilization [18].

The HRG A1042G SNP studied in this manuscript, which corresponds to a mutation in the His/Pro rich domain of HRG, was of interest based on previous knowledge indicating that this domain of HRG is important for the regulation of angiogenesis. Van Wildemeersch et al. [29] has presented evidence that a 35 amino acid long synthetic peptide corresponding to amino acids 330-364 in HRG located to the His/Pro rich domain, exhibited a Zn^2+^- dependent inhibitory effect on vessel formation both *in vitro* and *in vivo*. HRG 330-364 attached to heparin/heparan sulfate with equal capacity as full-length HRG. Peptides corresponding to other regions than the His/Pro-rich region of HRG-downstream of HRG 330-364 -lacked this inhibitory effect on endothelial cell migration and displayed a notably reduced heparin-binding capacity. The importance of this region in HRG was furthermore confirmed by Lee et al. [30]. They described that this region of HRG corresponds to the minimal anti-angiogenic active HRG fragment that is required for activation of endothelial cell focal adhesion, whereas changes in the region disrupt the cytoskeletal organization and the capacity of endothelial cells to assemble into vessel structures [31]. The latter is also in agreement with the concept that angiogenesis requires migration of endothelial cells [32] which is significant for implantation/placentation. In inadequately regulated placentation, pregnancy complications often develop. In summary, these studies suggest a connection between HRG and control of angiogenesis through release of the His/Pro rich region via a proteolytically regulated mechanism.

The exact pathway by which HRG exhibits its anti-angiogenic effect is not yet fully described; it seems nevertheless to be carried out through simultaneous multifactorial interaction. The His/Pro-rich region of HRG has been proposed to act through binding of cells at sites of tissue injury and thereby regulating the activity of degradative enzymes such as the plasminogen/plasmin system [33]. However, recent studies indicate that proteolysis mediated solely by plasmin is not sufficient to release the anti-angiogenic fragment of HRG due to the presence of an interdomain disulfide bridge connecting the NH2 domain to the His/Pro rich region [34]. Kassaar et al [34] with the use of x-ray crystallography has revealed the presence of an S-glutathionyl adduct at a cysteine residue (Cys185) of HRG, promoting the plasmin-mediated cleavage of the His/Pro fragment through a reduction/oxidation mechanism [34]. Although HRG has not been widely studied in the context of pregnancy or placental function, but mostly in tumors and carcinogenesis, similar conditions are thought to prevail in both situations. In conditions with limited supply of oxygen and nutrients (such as in tumors) treatment with recombinant human HRG led to reduced vascularization, increased apoptosis and decreased proliferation [32]. This was also confirmed by Juarez et al [20] who described that the anti-angiogenic effect of HRG, localized specifically to the His/Pro rich region, is mediated via increased activity of caspase-3-a and as a consequence via induced apoptosis of endothelial cells [20].

Another factor that stimulates the anti-angiogenic effect of HRG has been described to be blood platelets in an activated state. Activated maternal platelets were found in the lumen of the spiral arteries after histological examination of human placental tissue [35]. In an activated state, platelets release a variety of growth factors, cytokines and ions stored in granules. A local increase in Zn^2+^ concentration can induce a conformational change of the His/Pro rich domain and hence promote its binding to endothelial cells via heparan sulfate [12,20].

Despite the latter, one should always be cautious in interpreting genetic associations since there is always a possibility that the reported results represent a chance finding. It should however be noted that the background to the current hypothesis is based on a robust and widely accepted association between recurrent miscarriage and impaired implantation/placentation, due to inadequate angiogenesis [26]. One of the limitations with this study might be that a fraction of data is based on clinical information which is self-reported by the patients, in many cases years after the initial diagnosis, and we are thus obliged to accept that there might be a recall bias. On the other hand, this case-control study contains a very well defined and ethnically homogeneous population that strengthens the associations noted. Furthermore the ethnical distribution of the genetic variation in our study group is consistent with the distribution in HapMap-CEU population (rs2228243) [36], a population with similar geographic origin. Additional studies are though needed to further confirm our results as well as investigate the exact pathophysiological mechanism by which HRG variants are associated to recurrent miscarriage.

In conclusion, our results indicate an important role for HRG A1042G SNP in early pregnancy. It is shown that women who are heterozygous carriers of the HRG A1042G SNP suffer from recurrent miscarriage more seldom than homozygous carriers. Thus, analysis of the HRG A1042G SNP might be of importance for individual counseling regarding miscarriage.

## Abbreviations

ACOG: American college of Obstetricians and Gynecologists; ASRM: American society for reproductive medicine; ESHRE: European society of human reproduction and embryology; HRG: Histidine rich glycoprotein; NVOG: Dutch society of Obstetrics and Gynaecology; RCOG: Royal college of Obstetricians and Gynaecologists; SNP: Single nucleotide polymorphism; VEGF: Vascular endothelial growth factor.

## Competing interests

The authors declare that they have no competing interests.

## Authors’ contributions

EE performed the statistical analysis and interpretation of results, drafted, edited and reviewed the manuscript. KEL assisted in data analysis and in drafting the manuscript. HK, FH, KB, BML and LSS participated in the design of the study and acquisition of data. ASE participated in the design of the study, acquisition of data and in drafting the manuscript. AS assisted in data analysis, interpretation of data and in drafting the manuscript. ISP supervised the study, assisted in data analysis, interpretation of data and in drafting the manuscript. HÅ conceived of the study, participated in its design, assisted in the statistical analysis and interpretation of data, drafted, edited and reviewed the manuscript. All authors read and approved the final manuscript.

## References

[B1] Stirrat GM Recurrent miscarriage Lancet 1990 336 673 675 1975862 10.1016/0140-6736(90)92159-f

[B2] American College of Obstetricians and Gynecologists ACOG practice bulletin. Management of recurrent pregnancy loss. Number 24, February 2001 Int J Gynaecol Obstet 2002 78 179 190 12360906 10.1016/s0020-7292(02)00197-2

[B3] Jauniaux E Farquharson RG Christiansen OB Exalto N Evidence-based guidelines for the investigation and medical treatment of recurrent miscarriage Hum Reprod 2006 21 2216 2222 16707507 10.1093/humrep/del150

[B4] Royal College of Obstetricians and Gynaecologists (RCOG), S.A.C The Investigation and Treatment of Couples with Recurrent Miscarriage. 2011(Guideline No17.) 2014 http://www.rcog.org.uk/womens-health/clinical-guidance/investigation-and-treatment-couples-recurrent-miscarriage-green-top-

[B5] The Practice Committee of the American Society for Reproductive Medicine Definitions of infertility and recurrent pregnancy loss: a committee opinion Fertil Steril 2013 99 63 23095139 10.1016/j.fertnstert.2012.09.023

[B6] Goddijn M van den Boogaard E Steepers EA Erwich JJ Macklon NS Land JA Ankum WM The guideline ‘Recurrent miscarriage’ (first revision) of the Dutch Society for Obstetrics and Gynaecology Ned Tijdschr Geneeskd 2008 152 1665 1670 18714519

[B7] Blohm F Friden B Milsom I A prospective longitudinal population-based study of clinical miscarriage in an urban Swedish population BJOG 2008 115 176 182 18081599 10.1111/j.1471-0528.2007.01426.x

[B8] Rai R Regan L Recurrent miscarriage Lancet 2006 368 601 611 16905025 10.1016/S0140-6736(06)69204-0

[B9] Ole B HJ C Epidemiology of recurrent pregnancy loss Recurrent pregnancy loss 2007 1 London: Informa Healthcare 1 14

[B10] Daher S Mattar R Gueuvoghlanian-Silva BY Torloni MR Genetic polymorphisms and recurrent spontaneous abortions: an overview of current knowledge Am J Reprod Immunol 2012 67 341 347 22390536 10.1111/j.1600-0897.2012.01123.x

[B11] Su MT Lin SH Chen YC Genetic association studies of angiogenesis- and vasoconstriction-related genes in women with recurrent pregnancy loss: a systematic review and meta-analysis Hum Reprod Update 2011 17 803 812 21642294 10.1093/humupd/dmr027

[B12] Thulin A Ringvall M Dimberg A Karehed K Vaisanen MR Hamad O Wang J Bjerkvig R Nilsson B Pihlajaniemi T Akerud H Pietras K Jahnen-Dechent W Siegbahn A Olsson AK Activated platelets provide a functional microenvironment for the antiangiogenic fragment of histidine-rich glycoprotein Mol Cancer Res 2009 7 1792 1802 19903770 10.1158/1541-7786.MCR-09-0094

[B13] Poon IK Patel KK Davis DS Parish CR Hulett MD Histidine-rich glycoprotein: the Swiss Army knife of mammalian plasma Blood 2011 117 2093 2101 20971949 10.1182/blood-2010-09-303842

[B14] Nordqvist S Karehed K Hambiliki F Wanggren K Stavreus-Evers A Akerud H The presence of histidine-rich glycoprotein in the female reproductive tract and in embryos Reprod Sci 2010 17 941 947 20639474 10.1177/1933719110374366

[B15] Karehed K Wikstrom AK Olsson AK Larsson A Olovsson M Akerud H Fibrinogen and histidine-rich glycoprotein in early-onset preeclampsia Acta Obstet Gynecol Scand 2010 89 131 139 19878090 10.3109/00016340903295618

[B16] Natural Variations Database http://uniprot.org/uniprot/P04196/

[B17] The UniProt Consortium Activities at the Universal Protein Resource (UniProt) Nucleic Acids Res 2014 42 Database issue 191 198 10.1093/nar/gkt1140 PMC3965022 24253303

[B18] Nordqvist S Karehed K Stavreus-Evers A Akerud H Histidine-rich glycoprotein polymorphism and pregnancy outcome: a pilot study Reprod Biomed Online 2011 23 213 219 21665544 10.1016/j.rbmo.2011.04.004

[B19] Lindgren KE Karehed K Karypidis H Hosseini F Bremme K Landgren BM Skjoldebrand-Sparre L Stavreus-Evers A Sundstrom-Poromaa I Akerud H Histidine-rich glycoprotein gene polymorphism in patients with recurrent miscarriage Acta Obstet Gynecol Scand 2013 92 974 977 23672470 10.1111/aogs.12155

[B20] Juarez JC Guan X Shipulina NV Plunkett ML Parry GC Shaw DE Zhang JC Rabbani SA Mccrae KR Mazar AP Morgan WT Donate F Histidine-proline-rich glycoprotein has potent antiangiogenic activity mediated through the histidine-proline-rich domain Cancer Res 2002 62 5344 5350 12235005

[B21] Ibrahim MI Harb HM Ellaithy MI Elkabarity RH Abdelgwad MH First trimester assessment of pentraxin-3 levels in women with primary unexplained recurrent pregnancy loss Eur J Obstet Gynecol Reprod Biol 2012 165 37 41 22889492 10.1016/j.ejogrb.2012.07.016

[B22] Flicek P Amode MR Barrell D Beal K Billis K Brent S Carvalho-Silva D Clapham P Coates G Fitzgerald S Gil L Girón CG Gordon L Hourlier T Hunt S Johnson N Juettemann T Kähäri AK Keenan S Kulesha E Martin FJ Maurel T McLaren WM Murphy DN Nag R Overduin B Pignatelli M Pritchard B Pritchard E Riat HS H Ensembl 2014 Nucleic Acids Res 2014 42 Database issue D749 D755 24316576 10.1093/nar/gkt1196 PMC3964975

[B23] Hansson B Westerberg L On the correlation between heterozygosity and fitness in natural populations Mol Ecol 2002 11 2467 2474 12453232 10.1046/j.1365-294x.2002.01644.x

[B24] Gemmell NJ Slate J Heterozygote advantage for fecundity PLoS One 2006 1 e125 17205129 10.1371/journal.pone.0000125 PMC1762409

[B25] Laanpere M Altmae S Kaart T Stavreus-Evers A Nilsson TK Salumets A Folate-metabolizing gene variants and pregnancy outcome of IVF Reprod Biomed Online 2011 22 603 614 21507721 10.1016/j.rbmo.2011.03.002

[B26] Torry DS Leavenworth J Chang M Maheshwari V Groesch K Ball ER Torry RJ Angiogenesis in implantation J Assist Reprod Genet 2007 24 303 315 17616801 10.1007/s10815-007-9152-7 PMC3455012

[B27] Othman R Omar MH Shan LP Shafiee MN Jamal R Mokhtar NM Microarray profiling of secretory-phase endometrium from patients with recurrent miscarriage Reprod Biol 2012 12 183 199 22850470 10.1016/s1642-431x(12)60085-0

[B28] Krieg SA Fan X Hong Y Sang QX Giaccia A Westphal LM Lathi RB Krieg AJ Nayak NR Global alteration in gene expression profiles of deciduas from women with idiopathic recurrent pregnancy loss Mol Hum Reprod 2012 18 442 450 22505054 10.1093/molehr/gas017 PMC3431184

[B29] Vanwildemeersch M Olsson AK Gottfridsson E Claesson-Welsh L Lindahl U Spillmann D The anti-angiogenic His/Pro-rich fragment of histidine-rich glycoprotein binds to endothelial cell heparan sulfate in a Zn2 + -dependent manner J Biol Chem 2006 281 10298 10304 16436387 10.1074/jbc.M508483200

[B30] Lee C Dixelius J Thulin A Kawamura H Claesson-Welsh L Olsson AK Signal transduction in endothelial cells by the angiogenesis inhibitor histidine-rich glycoprotein targets focal adhesions Exp Cell Res 2006 312 2547 2556 16769050 10.1016/j.yexcr.2006.04.022

[B31] Blank M Shoenfeld Y Histidine-rich glycoprotein modulation of immune/autoimmune, vascular, and coagulation systems Clin Rev Allergy Immunol 2008 34 307 312 18219588 10.1007/s12016-007-8058-6

[B32] Olsson AK Larsson H Dixelius J Johansson I Lee C Oellig C Bjork I Claesson-Welsh L A fragment of histidine-rich glycoprotein is a potent inhibitor of tumor vascularization Cancer Res 2004 64 599 605 14744774 10.1158/0008-5472.can-03-1941

[B33] Wakabayashi S Koide T Histidine-rich glycoprotein: a possible modulator of coagulation and fibrinolysis Semin Thromb Hemost 2011 37 389 394 21805445 10.1055/s-0031-1276588

[B34] Kassaar O McMahon SA Thompson R Botting C Naismith JH Stewart AJ Crystal structure of histidine-rich glycoprotein N2 domain reveals redox activity at an interdomain disulfide bridge: impications for angiogenic regulation Blood 2014 123 1948 1954 24501222 10.1182/blood-2013-11-535963 PMC3962167

[B35] Sato Y Fujiwara H Konishi I Role of platelets in placentation Med Mol Morphol 2010 43 129 133 20857260 10.1007/s00795-010-0508-1

[B36] Database of Single Nucleotide Polymorphisms (dbSNP) http://www.ncbi.nlm.nih.gov/SNP/

